# PVDF-HFP-based polymer inclusion membrane functionalized with D2EHPA for the selective extraction of bismuth(III) from sulfate media

**DOI:** 10.1038/s41598-024-62401-8

**Published:** 2024-05-21

**Authors:** Davood Kazemi, Mohammad Reza Yaftian

**Affiliations:** Department of Chemistry, Faculty of Science, University of Zanjan, Zanjan, 45371-38791 Iran

**Keywords:** PVDF-HFP, D2EHPA, Extraction, Polymer inclusion membrane (PIM), Bismuth(III), Masking agent, Analytical chemistry, Environmental sciences

## Abstract

This study is the first application of a PVDF-HFP-based polymer inclusion membrane incorporating the poly(vinylidene fluoride-co-hexafluoropropylene) (PVDF-HFP) and di(2-ethylhexyl)phosphoric acid (D2EHPA) as the base polymer and extractant for the extraction of bismuth(III), respectively. It is demonstrated that the PIM comprised of 60 wt% PVDF-HFP and 40 wt% D2EHPA is the most effective in the extraction of bismuth(III) from feed solution containing 20 mg L^−1^ bismuth(III) and 0.2 mol L^−1^ sulfate adjusted to pH 1.4. The extracted bismuth(III) ions are back-extracted quantitatively to the receiving solution containing 1 mol L^−1^ sulfuric acid. The stoichiometry experiments reveal that the Bi: D2EHPA ratio in the bismuth(III) extracted complex is 1:6, and D2EHPA is dimer. Moreover, it is shown that the studied PIM has high selectivity in the extraction of bismuth(III) over other interfering ions such as Mo(VI), Cr(III), Al(III), Fe(III), Ni(II), Zn(II), Cd(II), Co(II), Cu(II), and Mn(II). The interference of Fe(III) is also eliminated by masking with fluoride, leading finally to a nearly pure extraction of bismuth(III).

## Introduction

Bismuth, with its less toxic nature, is one of the technology-critical elements and is widely used in various medical and industrial (e.g., cosmetics, alloys, semiconductors, pharmaceutics, and ceramics) productions^1–5^. However, the abundance of bismuth minerals is low, and bismuth is mainly acquired as the by-product of copper, tin, and lead–zinc production factories^6,7^. Increasing the applications of bismuth has made the waste and wastewater containing bismuth to be considered as the favorable bismuth supplies^8,9^. So developing new separation methods for the recovery of bismuth from waste or wastewater has achieved great attention in the few last decades. Typically, bismuth-containing materials are leached in acidic solutions. The solvent extraction methods are conventional methods for the recovery of bismuth from these acidic solutions. Several extractants including amines^10^, organophosphorus extractants^11–14^, and sulfur-containing extractants^15,16^ have been employed for the extraction of bismuth from various solutions. Medjahed et al.^17^ employed aminododecyldiphosphonic acid (ADDMDPA) for the extraction of bismuth(III) from nitrate medium. The solvent extraction of bismuth(III) from the nitric acid medium using the synergistic effect of 2-ethylhexylphosphonic acid mono-(2-ethylhexyl) ester (HEHEHP) and 2,2ʹ-bipyridyl (bipy) was investigated by Song et al.^13^. In addition, Jiang et al. reported the synergistic effect of sec-octylphenoxy acetic acid and 1,10-phenanthroline in the recovery of bismuth(III) from a chloride medium^18^.

It has been shown that solvent extraction techniques have an appropriate separation ratio. However, toxic and flammable organic solvents are costly as well as can cause environmental pollution^19^. Recently, membrane-based techniques have gained attraction due to their scale-up potential. They can considerably reduce or eliminate the consumption of organic solvents^20^. A variety of configurations were reported for the recovery of bismuth by using bulk^15^, emulsion^21,22^, and supported liquid membranes^6,23,24^. The bulk liquid membranes (BLMs) have a low ratio of membrane to interfacial surface areas of the aqueous phase, leading to a low mass transfer rate. The emulsion liquid membranes (ELMs) have also a great emulsion breakage. These drawbacks prohibited BLMs and ELMs as the proper methods for replacing solvent extraction. Supported liquid membranes (SLMs) provided approximately large interfacial surface areas and high mass transfer rates. However, the poor stability of SLM hindered large-scale and industrial applications.

Polymer inclusion membranes (PIMs) require only minimal quantities of organic solvent and extractant^25^. The PIMs can be feasibly created by eco-friendly solvents^26^. The PIMs are generally stable and versatile over SLMs due to the entanglement of the membrane liquid phase (extractant and plasticizer) within the strands of the utilized base polymer.

PIMs are a type of liquid membrane that can be adjusted for the extraction of metal ions and support separation based on chemical reactions^27^. PIMs are easily formed by the solvent casting technique by mixing a base polymer and an extractant. Plasticizers can be also added to the formulation of PIMs. Plasticizers are used to make the membrane softer and more flexible, retain the metal species, and subsequent transportation of these species across the membrane^28^. The plasticizer penetrates between polymer molecules and neutralizes the polar groups of the polymer with its own polar groups or merely increases the distance between the polymer molecules and hence reduces the strength of the intermolecular forces^29^. However, in some cases, membrane production can take place without plasticizers because some extractants can also act as plasticizers (i.e. D2EHPA), and their use in PIM's composition does not necessarily require the addition of plasticizers^19,25,30^. However, the PIMs without plasticizers are more favorable because they are simply fabricated and also cheaper. Selection of the brad base polymers, such as poly(vinylidene fluoride-co-hexafluoropropylene) (PVDF-HFP)^31^, cellulose triacetate (CTA)^32^, polyvinylidene fluoride (PVDF)^33^, and polyvinyl chloride (PVC)^34^ makes it possible to prepare diverse PIMs with different characteristics. The PVDF-HFP, the base polymer in this work, has motivated considerable interest owing to its higher hydrophobicity, better thermal and mechanical characteristics, and excellent stability in the presence of strong acids^31^. The characteristics of PIMs can be effectively controlled by utilizing blend polymers as the base polymer. The cross-linked PIMs (CL-PIMs) were a new generation of PIMs. They exploited the cross-linked base polymer and showed high stability and performance^35^. However, it has been shown that the preparation of CL-PIMs is complicated.

The extractant is responsible for forming either a complex or an ion pair with the target ion^36^. There are a lot of compounds that have the potential to act as an extractant in PIMs. An appropriate extractant is selected based on the properties of the target ion. When a target ion is a cation, such as Bi^3+^, the appropriate extractants can be either acidic or chelating extractants. D2EHPA, used in this work, is an acidic commercial extractant that can exchange hydrogen ions with other cations existing in the aqueous feed solution^37^.

Recently, it has been shown that the CTA-based PIM comprised of 40 wt% CTA, 25 wt% trioctylamine (TOA), and 35 wt% tributyl phosphate (TBP) has great potential for the quantitative extraction of bismuth(III) as anionic chloro-complex form from its chloride solutions. However, due to the low stability of CTA-based PIM, it was used only in four consecutive extraction/back-extraction experiments^38^. Kazemi et al.^39,40^ showed the extraction-recovery of bismuth(III) as cationic form from its sulfate solution by using PVC-based PIM comprised of 50/50 wt% PVC/D2EHPA. It was shown that PVC-based PIM had suitable stability. They showed that the optimized PIM composed of 50/50 wt% PVC/D2EHPA could be used at least in 15 extraction/back-extraction cycles and in 10 consecutive transport experiments. It was proved that the selectivity of PVC-based PIM in transport experiments is more efficient than in extraction experiments. Meziani et al.^41^ employed two PIMs composed of CTA/Aliquat 336/NPOE and CTA/Cyphos® 101/NPOE for the recovery of bismuth from its chloride solutions. They showed that the recovery of bismuth is independent of the cationic part of ionic liquids and also reported that both PIMs were able to quantitatively recover bismuth ions. PIM composed of CTA/Cyphos® 101/NPOE was more stable than the other one so it was used in 7 cycles of the experiment. However, their selectivity studies demonstrated that Sb(III) and Pb(II) were co-transported with bismuth ions without affecting the effectivity of the PIM in bismuth ion extraction.

In this work, we investigated the self-plasticized PVDF-HFP-based PIM for the selective extraction of bismuth(III) from sulfate media by using PIMs composed of PVDF-HFP as the base polymer and D2EHPA as the extractant. We exploited Fourier transform infrared (FT-IR), atomic force microscopy (AFM), scanning electron microscopy (SEM), thermogravimetric analysis (TGA), contact angle (CA), and stress–strain curves to characterize the interactions, morphology, and mechanical properties of the studied PIM. The efficiency of PVDF-HFP-based PIM for the extraction of bismuth(III) over other competing ions such as Mo(VI), Fe(III), Cr(III), Al(III), Ni(II), Zn(II), Cd(II), Co(II), Cu(II), Mn(II) was investigated. Our results showed that bismuth(III) was efficiently extracted by using the studied PIM. It was shown that PVDF-HFP-based PIM has great stability in consecutive extraction/back-extraction experiments at least in 15-cycle extraction/back-extraction experiments without no decreasing performance. We demonstrated that PVDF-HFP acts like a non-polar solvent in which D2EHPA was dispersed in the PVDF-HFP matrix in dimeric form. Moreover, it was illustrated that the possible structure of Bi-D2EHPA can be obtained from extraction stoichiometry results.

## Experimental

### Chemical and reagents

The chemical reagents utilized to cast the PIMs were Poly(vinylidene fluoride-co-hexafluoropropylene) (PVDF-HFP) pellets (Castle Hill, NSW, Australia), di(2-ethylhexyl)phosphoric acid (D2EHPA) (≥ 97%, Sigma-Aldrich, MO, USA), and tetrahydrofuran (THF) (≥ 99.8, Merck, Darmstadt, Germany). The investigated plasticizers were 2-nitrophenyl octyl ether (NPOE) (99%, Sigma Aldrich, Hamburg, Germany), tris(2-ethylhexyl)phosphate (TEHP) (≥ 97%, Sigma Aldrich, Germany), bis(2-ethylhexyl) phthalate (DEHP) (99.5%, Fluka, Buchs, Switzerland), 1-tetradecanol (≥ 98%, Merck, Germany),Tri-n-butyl phosphate (TBP) (> 98%, Merck, Darmstadt, Germany), and phthalic acid dibutyl ester (DBP) (99%, Sigma-Aldrich, USA).

Sulfuric acid (98%, Neutron, Iran), Bi(NO_3_)_3_ 0.5H_2_O (> 99%, Alfa Aesar, ACS), Fe(NO_3_)_3_·9H_2_O (≥ 98%, Alpha Chemika, Maharashtra, India), ZnSO_4_·7H_2_O (≥ 99%, Sigma-Aldrich, MO, USA), Ni(NO_3_)_2_ 0.6H_2_O (≥ 99%, Carlo Erba, Italy), Cu(NO_3_)_2_ 0.3H_2_O (≥ 99.5%, Avantor, Gliwice, Poland), Na_2_SO_4_ (≥ 99%, Neutron, Iran), AlCl_3_ (≥ 99%), CrCl_3_ (99%), Co(NO_3_)_3_·6H_2_O (≥ 98%), Mn(NO_3_)_2_. 4H_2_O (≥ 98%), NaF(≥ 99.5%), NaOH (≥ 98%), and CdSO_4_ 0.8H_2_O (≥ 98%,), all from Merck (Darmstadt, Germany). All aqueous solutions were prepared in ultrapure water (resistivity ≥ 18.2 MΩ cm, Zolalan, m-uv-3^+^, Iran).

### Instruments

The magnetic stirrer (IKA, Warsaw, Poland) and thermoregulatory (Org Mp5, Julabo, Germany) were used in the PIM preparation. An orbital shaker (HLB501, Behsan, Iran) was employed to agitate the plastic jars that contained feed and receiving solutions. The aqueous solution pH measurements were made using a Metrohm analyzer (780 Herisau, Switzerland) with a pH probe (Metrohm, Switzerland). The concentration of all metal ions was measured with an atomic absorption spectrometer (AAS) (AA-7800, Shimadzu, Japan). A Multiskop goniometer (Optrel, Germany) was used to determine the water contact angle of PVDF-HFP film and selected PIM. The sessile drop method was used to measure the contact angle of the prepared PVDF-HFP film and optimized PIM. An ultrapure water drop of 3 µL was deposited on the membrane surface. For each sample, the contact angle between the water drop and the membrane surface was determined by the CAM software. The average of five drops at different locations of membranes was calculated. Thermal analyses (TGA and DTGA) of the membranes were performed in the nitrogen atmosphere using a TGA analyzer (Q50, TA Instruments, USA) at a heating rate of 10 °C min^−1^. The morphology of selected PIM and pure PVDF-HFP film was investigated by scanning electron microscopy (SEM) (JSM-7001F, Japan) and atomic force microscopy (AFM) (Oxford Instruments, USA). To obtain a clean cross-sectional image suitable for analyzing the internal PIM morphology, the samples were prepared by freezing the membrane with liquid nitrogen followed by rapid breaking. All samples underwent sputter-coating with gold before microscopic analysis. The FT-IR spectra of D2EHPA, PVDF-HFP film, and optimized PIM were obtained using FT-IR spectroscopy (Thermo Fisher Scientific Inc, Nicolet is10, USA) according to the Attenuated Total Reflectance (ATR) technique in a wavenumber range of 400–4000 cm^−1^.

The mechanical properties of optimized PIM and blank PVDF-HFP film were tested using 1 cm wide and 3 cm long samples. A digital force gauge (Ametek, Lloyd LS5, USA) with a maximum tension of 100 N and length accuracy of ± 0.01 mm connected to a computer was used to perform the measurements. The Measurements were conducted at a strength rate of 10 mm s^−1^. The thickness of all PIMs was measured with a digital micrometer (Werka, China). A homemade casting knife was used in the preparation of the membranes. The schematic diagram of the casting knife with its dimensions is illustrated in Fig. S1.

### Preparation of the PIMs

To create PVDF-HFP-based PIMs, the membrane components were dissolved in THF (5 mL of THF per 0.5 g of base polymer^42^) under magnetic stirring for 2 h at ambient temperature and 2 h at 42 °C. The obtained casting solution was poured onto a flat glass plate by using a homemade casting knife. Then, the glass plate was covered with an aluminum tray to allow the slowly evaporate THF overnight. Finally, flat-sheet PIMs were peeled off from the glass plate. The membranes were then cut into circular segments with a diameter of 3.5 cm by using a steel punch. The average mass and thickness of circular pieces of the optimized membrane (60/40 wt% PVDF-HFP/D2EHPA) were 49 ± 2.8 mg and 52 ± 1.9 μm, respectively.

### Membrane extraction and back-extraction experiments

To perform the extraction experiments, the PIM segments were immersed in plastic jars containing 50 mL of feed solution. Then, the plastic jars were shaken at 200 rpm on a platform orbital shaker. 0.5 mL samples were taken of the feed solution at regular time intervals. The feed solution was composed of 20 mg L^−1^ bismuth(III) and 0.2 mol L^−1^ sodium sulfate adjusted to pH 1.4. To conduct the back-extraction experiments, the loaded PIM segments were placed into the plastic jars containing 50 mL of 1 mol L^−1^ sulfuric acid stripping solutions and shaken at 200 rpm on a platform orbital shaker for 10 min. The concentration of metal ions was measured in all extraction and back-extraction samples by AAS. The extraction and back-extraction experiments were performed three times at ambient temperature.

## Results and discussion

### Shaking rate optimization

**Figure 1:**
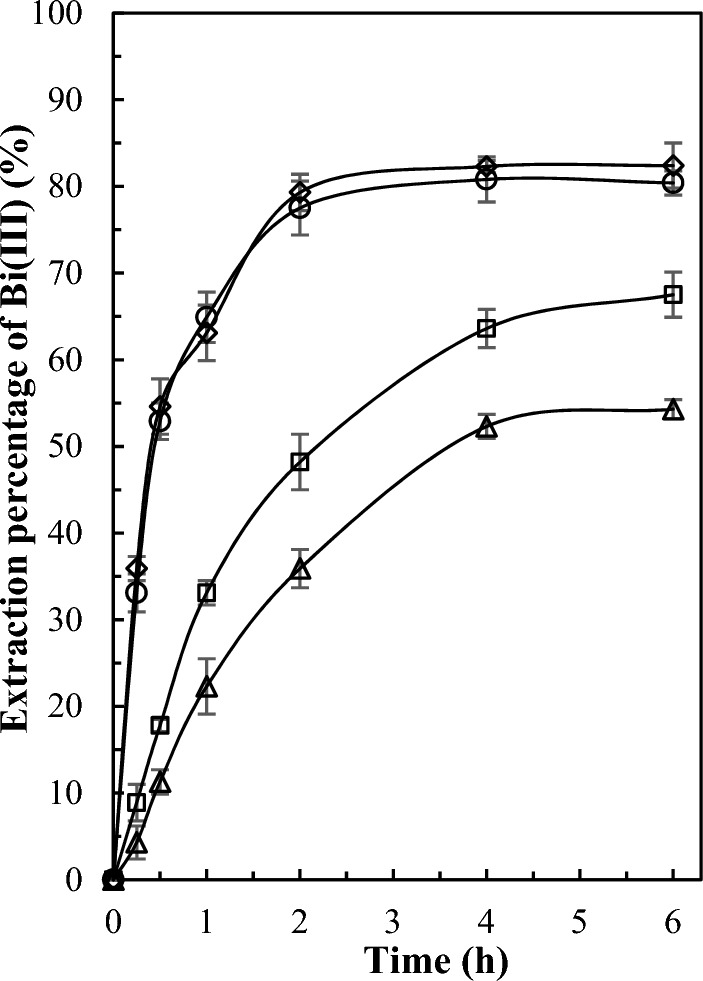
The influence of shaking rate [30 (open triangle), 100 (open square), 200 (open circle), 400 (open diamond) rpm] on the extraction of bismuth(III) by selected PIM (60/40 wt% PVDF-HFP/D2EHPA). Experimental conditions: 50 ml of aqueous feed phase containing 20 mg L^−1^ of bismuth(III) and 0.2 mol L^−1^ sulfate adjusted to pH 1.4. Error bars ± standard deviation (SD) (n = 3).

To enhance the efficiency of extraction processes using PIMs, the elimination of mass transfer effects is crucial. In this regard, the effect of the shaking rate on the extraction of bismuth(III) was investigated by using different shaking rates in the range of 30–400 rpm. The variation of extraction percentage of bismuth(III) as a function of time in different shaking rates is presented in Fig. 1. The obtained results demonstrated that the extraction rate increases with the increase in stirring rate up to 200 rpm, after which further increment in stirring rate does not significantly affect the extraction rate. This is due to an increase in stirring rate reducing the thickness of the stagnant diffusion layer, consequently leading to a higher extraction rate. Moreover, at a shaking rate of 200 rpm and beyond, the extraction process was governed by extraction kinetics and mass transfer processes within the PIM, rendering the extraction rate independent of the shaking rate^43^. Based on the obtained results, a shaking rate of 200 rpm was consistently employed in all subsequent experiments.

### PIMs screening experiments

In order to optimize the concentration of the extractant, a series of membranes comprised of PVDF-HFP with weight percentages between 30 and 50 of D2EHPA were prepared. As the extractant content increased, the flexibility of the membranes also increased. All the prepared membranes were transparent and flexible. However, the membrane containing 50 wt% extractant did not possess sufficient mechanical stability and therefore was not suitable for use. The prepared membranes were used in the extraction experiments of bismuth(III) from the sulfate medium.

**Figure 2:**
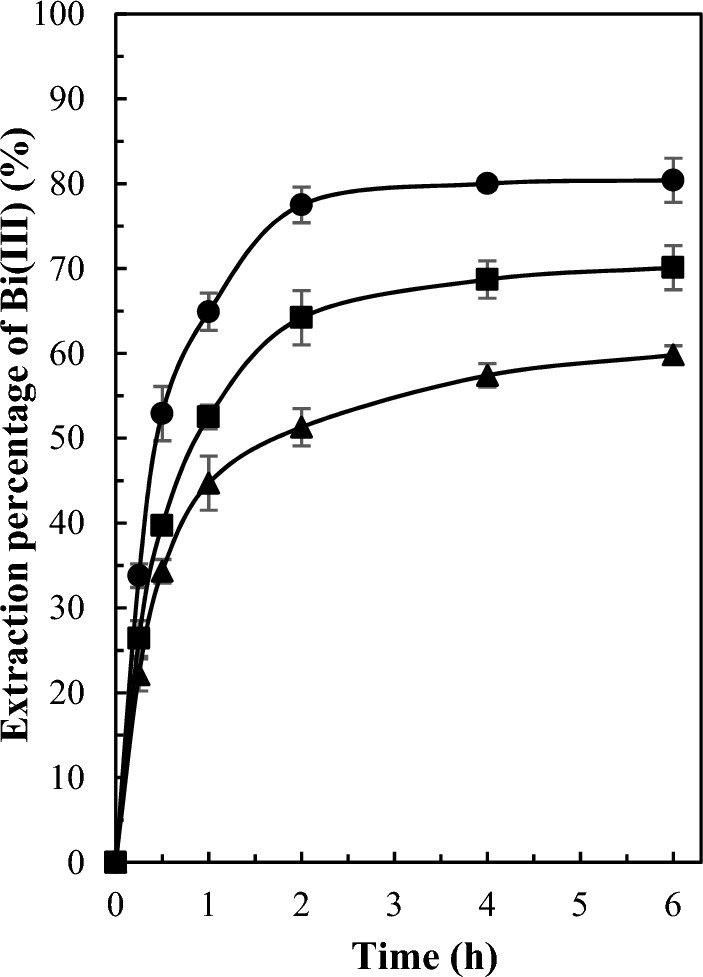
Extraction of bismuth(III) by PVDF-HFP/D2EHPA PIMs with different compositions (60/40 (filled black circle), 65/35 (filled black square), and 70/30 (filled black triangle) wt% PVDF-HFP/D2EHPA). Experimental conditions: 50 ml of aqueous feed phase containing 20 mg L^−1^ of bismuth(III) and 0.2 mol L^−1^ sulfate adjusted to pH 1.4. Shaking rate: 200 rpm. Temperature 25 ± 1 °C. Error bars ± standard deviation (SD) (n = 3).

The variation of extraction percentage of bismuth(III) as a function of time using the investigated PIMs is illustrated in Fig. 2. As expected, the extraction percentage increases with an increase in the weight percentage of the D2EHPA extractant. The highest extraction percentage of bismuth(III) was observed after 4 h using a membrane comprised of 60/40 wt% PVDF-HFP/D2EHPA. Hence, the membrane with a composition of 60/40 wt% PVDF-HFP/D2EHPA was selected as the optimal membrane and was employed in all subsequent experiments.

### Effect of the plasticizer type on the PIMs properties

Although the prepared membranes without plasticizers exhibited suitable and acceptable properties for bismuth extraction, a study was performed to investigate the effect of plasticizers on the Polymer inclusion membrane properties. For this purpose, membranes were prepared with a composition of 55 wt% PVDF-HFP, 40 wt% D2EHPA, and 5 wt% of plasticizers such as DBP, 1-TD, TBP, NPOE, DEHP, or TEHP. All prepared membranes, regardless of the type of plasticizer used, exhibited suitable physical and mechanical properties and were used in bismuth extraction experiments from the sulfate medium. The performance of these membranes was compared to the 60/40 wt% PVDF-HFP/D2EHPA membrane without a plasticizer. The evolution of the extraction percentage of bismuth(III) as a function of time for PIMs based on studied plasticizers is illustrated in Fig. S2. As can be seen, the increase in plasticizer content did not have a significant effect on the extraction efficiency of bismuth(III). The obtained results indicated that the membrane without plasticizer composed of 60/40 wt% PVDF-HFP/D2EHPA was a suitable PIM for further study.

### PIM characterization

#### Hydrophobic/hydrophilic character

The hydrophilicity of the membrane surfaces was investigated by using contact angle measurements (Fig. S3). The contact angle value for the pure PVDF-HFP membrane was determined to be 94.0° ± 1.8°, while the optimized membrane containing 40 wt% D2EHPA exhibited a contact angle of 67.8° ± 2.1°. These results indicated that the presence of the extractant significantly decreased the surface hydrophobicity of the membrane. This can be attributed to the polar functional groups present in the extractant at the membrane surface. In the study conducted by Fontàs et al.^44^, it was discovered that the decrease in water contact angle can be attributed to the increase in concentration of the hydrophilic groups in PIM. This phenomenon is observed when there is an increase in the content of an acidic extractant (Lasalocid A) or ionic liquid (Aliquat 336). Such reduction in the contact angle with increasing D2EHPA content in the PVDF-HFP-based PIMs has been also reported by Wang et al.^45^.

#### Selected PIM morphology

**Figure 3:**
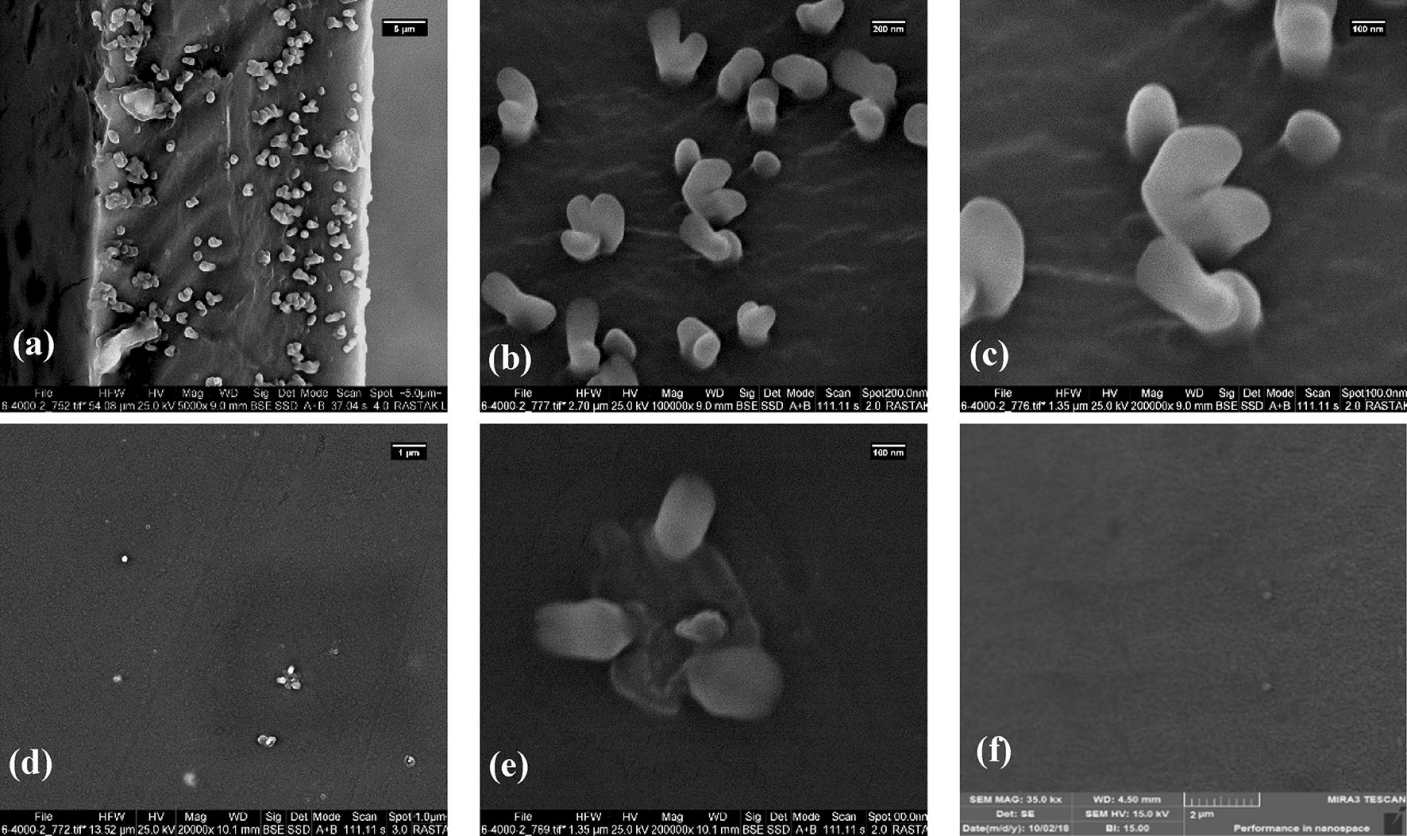
SEM images of the (**a**, **b**, **c**) cross-section of the selected PIM, (**d**, **e**) surface of the selected PIM, and (**f**) surface of the blank PVDF-HFP film.

SEM images of the surface and cross-section of the pure PVDF-HFP membrane and the optimized membrane containing 60 wt% D2EPA are shown in Fig. 3. As observed, the pure PVDF-HFP film exhibited smooth surface. The SEM images of the cross-section and surface of the optimized membrane revealed the presence of small droplets of the extractant. The presence of D2EHPA in the cross-section of the optimized PIM revealed the formation of continuous liquid domains (pathways) through the PIM. It can be assumed that these liquid domains in the PIMs act as "liquid pores", facilitating the transport process through the PIMs^46^.

The EDS analysis was conducted to investigate the presence of bismuth in the loaded membrane. The EDS result for the loaded PIM with bismuth(III) is illustrated in Fig. S4a. As can be observed, the F and C elements are attributed to the base polymer (PVDF-HFP), while the P, O, and C correspond to the extractant (D2EHPA). Additionally, the presence of the bismuth element in Fig. S4a was the main characteristic feature of the EDS analysis of the loaded PIM that revealed the optimized PIM extracted the bismuth ions from the feed solution.

Furthermore, the distribution of extractants in the optimized PIM after loading by bismuth (III) was also studied with EDS mapping and the results are presented in Fig. S4b. The EDS mapping of elements F and C represents the PVDF-HFP, while P, O, C, and Bi represent the D2EHPA and bismuth ions, respectively.

AFM images for the blank PVDF-HFP membrane and the optimized membrane are shown in Fig. S5. The surface roughness for the pure PVDF-HFP membrane and the optimized membrane containing 40 wt% D2EPA were 19.8 nm and 8.7 nm, respectively. Based on this, it can be observed that the presence of the D2EPA in the membrane matrix reduces its wrinkling and folding compared to the blank PVDF-HFP film.

The obtained AFM results are in good agreement with Ladewig and Al-Shaeli^47^. They reported the morphology of the membranes is highly dependent on the preparation method, and that dense membranes are formed when the solvent evaporation method is utilized.

#### TGA and DTGA analysis

**Figure 4:**
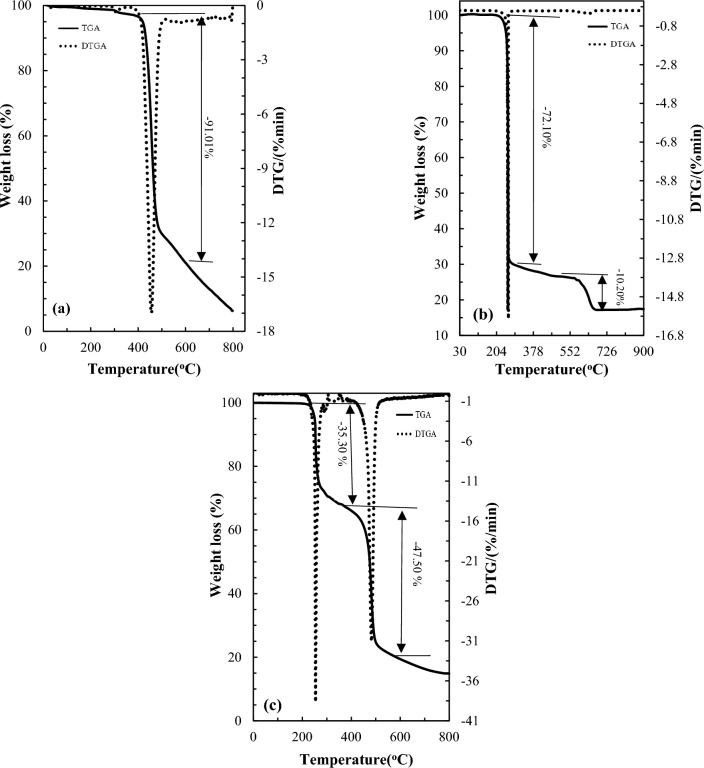
TGA and DTG curves of the (**a**) blank PVDF-HFP film and (**b**) D2EHPA, and (**c**) selected PIM (60/40 wt% PVDF-HFP/D2EHPA).

The thermal stability of the optimized membrane was investigated using thermogravimetric analysis (TGA) and differential thermogravimetric analysis (DTGA) as illustrated in Fig. 4. In the blank PVDF-HFP film, there is a weight loss region indicating the degradation of PVDF-HFP occurred in only one stage at temperatures close to 450 °C with a total mass loss of approximately 91%. The TGA thermogram of the D2EHPA shows that D2EHPA was decomposed in two weight loss stages. The first degradation of D2EHPA was observed at temperatures close to 215 °C with a total mass loss of approximately 75%, while the remaining mass loss occurred at 600 °C. This observation is consistent with other TGA studies involving D2EHPA under similar experimental conditions^48,49^.

However, the results revealed that the optimized membrane containing 40 wt% D2EPA decomposed in two weight loss stages. The first stage occurs at 254 °C with a weight loss of 35.3%, and the second degradation stage occurs at 482 °C with a weight loss of 47.5%. Both weight loss regions can be attributed to the degradation of the D2EHPA and the PVDF-HFP polymer. At last, the last observed weight loss corresponds to the decomposition and conversion of the remaining membrane into carbon dioxide and volatile hydrocarbons.

The comparison of the TGA results between the pure PVDF-HFP membrane and the optimized membrane revealed that the thermal degradation of the PVDF-HFP in the optimized membrane occurs at a lower temperature compared to the pure PVDF-HFP membrane. The presence of D2EHPA facilitates the degradation of PVDF-HFP, leading to the observed effect. The lower temperature degradation of PVDF-HFP in the optimized membrane suggesting that it has interact with D2EHPA by the weak interactions, such as van der Waals forces and hydrogen bonds. This conclusion confirms the FTIR results.

#### Tensile tests

The mechanical properties of the membrane were evaluated using stress–strain curves (Fig. S6a). The results depicted in Fig. S6a revealed that the presence of the D2EHPA in the optimized membrane leads to a reduction in tensile strength and an increase in elongation properties of the membrane. This effect demonstrates the plasticizing effect of the D2EHPA.

To investigate the mechanical properties of PIM containing more than 40 wt% D2EHPA, the stress–strain curve of PIM composed of 50/50 wt% PVDF-HFP/D2EHPA was obtained and compared with the stress–strain curve of optimized PIM, as shown in Fig. S6b. The obtained result revealed that the tensile strength decreased and the elongation properties increased in the PIM composed of 50/50 wt% PVDF-HFP/D2EHPA. These results revealed that the PVDF-HFP-based PIMs containing 50 wt% D2EHPA haven’t sufficient mechanical stability and therefore were not suitable for use.

#### FT-IR spectroscopy measurements

**Figure 5:**
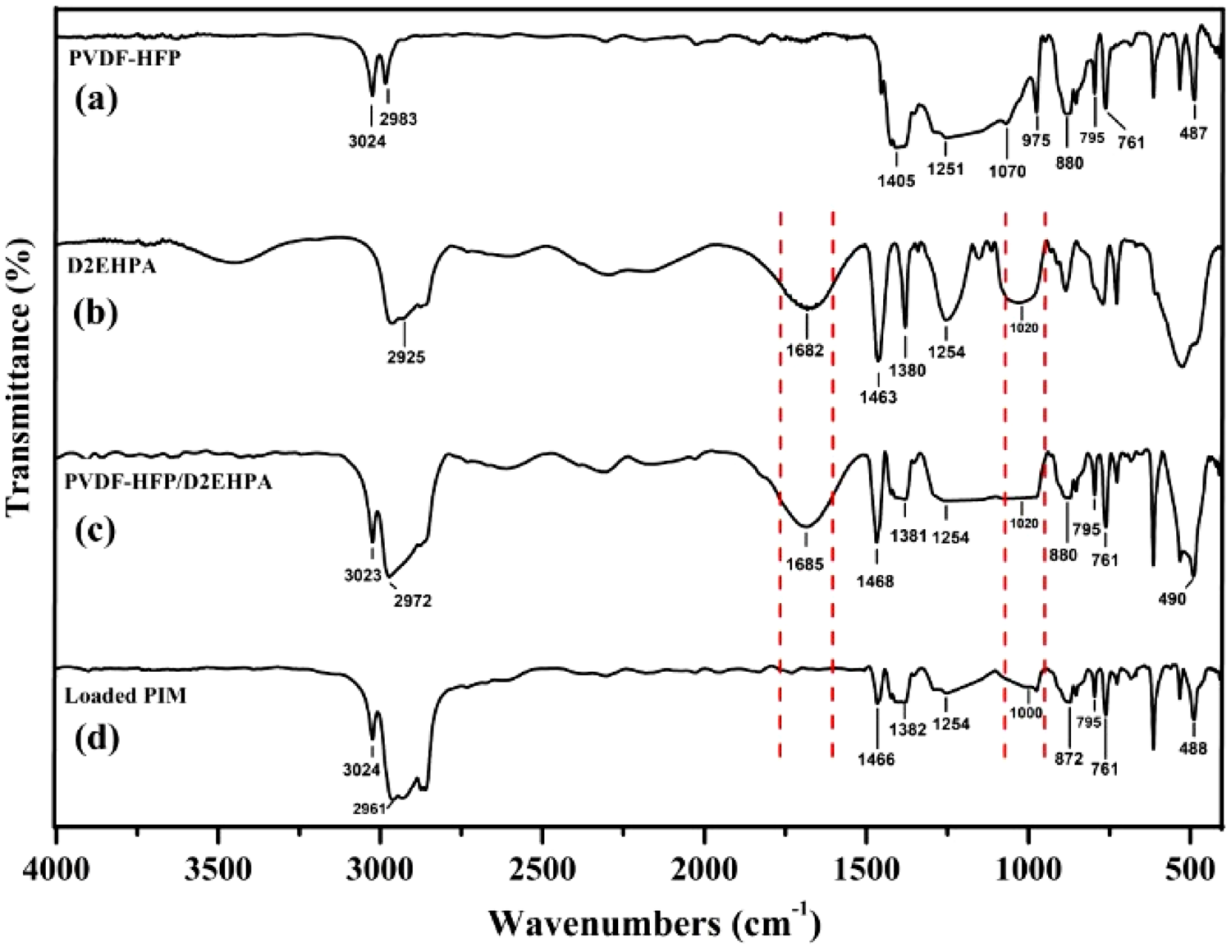
FT-IR spectra of the (**a**) blank PVDF-HFP film, (**b**) D2EHPA, and (**c**) selected PIM (60/40 wt% PVDF-HFP/D2EHPA) (**d**) selected PIM loaded with bismuth(III).

FT-IR spectroscopy was performed to investigate the possible interactions and nature of interactions between PVDF-HFP and the D2EPA in the optimized membrane. For this purpose, the FT-IR spectra were recorded for a pure PVDF-HFP membrane, D2EHPA, and the optimized membrane (Fig. 5). In the FT-IR spectrum of the pure PVDF-HFP membrane (Fig. 5a), the absorption band at 1405 cm^−1^ corresponds to the stretching and bending vibrations of C–F and CH_2_ groups, which overlap with each other. Additionally, the band at 975 cm^−1^ is attributed to the bending vibrations of C–H groups. The bands at 880 cm^−1^ and 1070 cm^−1^ are assigned to the bending and stretching vibrations of C–C groups. The –CF_2_ bending and stretching peaks are observed at 487 cm^−1^, 761 cm^−1^, and 1251 cm^−1^, while the peak at 795 cm^−1^ corresponds to the stretching vibrations of CF_3_ groups. The absorption bands at 975 cm^−1^, 2983 cm^−1^, and 3024 cm^−1^ are assigned to the bending and stretching vibrations of C–H groups. In the FT-IR spectrum of D2EHPA (Fig. 5b), the band at 1020 cm^−1^ corresponded to the P–O–C and P–O–H functional groups, while the band at 1254 cm^−1^ was assigned to the P=O functional group. The stretching vibration mode of O–H was significantly lowered and observed at 1682 cm^-1^ due to the occurrence of hydrogen bonding between D2EPA molecules. The stretching vibration mode of alkyl groups was observed at 2925 cm^−1^, while the –CH_2_ deformation modes were observed at 1380, and 1463 cm^−1^.

The presence of bands of the PVDF-HFP and D2EHPA was the main characteristic feature of the FT-IR spectrum of optimized PIM. In the FT-IR spectrum of the selected PIM (Fig. 5c), the bands located at 490, 761, 795, 1254, 880, 2972, and 3023 cm^−1^ were also observed in the spectra of PVDF-HFP, and the bands at 1254, 1380, 1468, and 1685 cm^−1^ were assigned to the D2EHPA presence. Additionally, three bands at 1020, 1070, and 2925 cm^−1^ represent the overlapping peaks of PVDF-HFP and D2EHPA. The FT-IR spectrum of the optimized membrane reveals the presence of functional groups from both PVDF-HFP and D2EHPA. This indicates that there is no significant chemical interaction between the membrane components. Instead, the physical stability of the membrane is attributed to weak interactions, such as van der Waals forces and hydrogen bonds.

In the FT-IR spectrum of the loaded membrane (Fig. 5d), the band at 1685 cm^−1^ corresponded to the O–H of D2EHPA was disappeared, while the band at 1020 cm^−1^ attributed to the P–OH and P–OC of D2EHPA was shifted to 1000 cm^-1^. The disappearance of the O–H vibration of D2EHPA indicated that D2EHPA protons were separated upon complex formation and bonded to bismuth ions. This result is supported by the shifting of P-O vibrations to lower frequencies than those of free D2EHPA.

### Effect of pH

**Figure 6:**
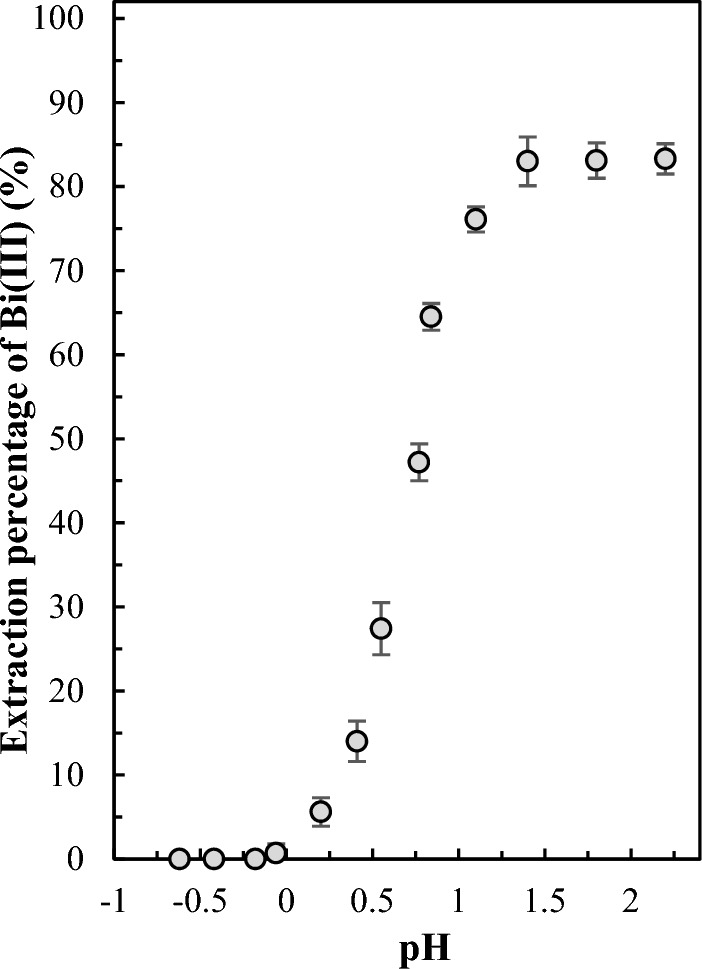
pH isotherm of selected PIM. The experimental conditions are as in Fig. 2.

Due to the fact that D2EHPA is an acidic extractant, the pH of the feed phase is expected to play a decisive role in the extraction process. To assess the effect of this parameter, the extraction experiments were conducted using feed solutions with different pH values ranging from − 0.6 to 2.2. The variations of extraction percentage of bismuth(III) as a function of pH are presented in Fig. 6. The obtained results revealed that initially, the extraction percentage increased with increasing feed solution pH from − 0.6 to 1.4 and then it plateaued at nearly 80% within the pH range of 1.4–2.20. pH values higher than this range were not studied due to the precipitation of bismuth. Since D2EHPA exhibits high selectivity for bismuth at low pH, a pH of 1.4 was selected as the optimum pH, and in all subsequent experiments, the pH of the feed solutions was adjusted to 1.4. Due to the feed solution's high acidity and low bismuth(III) concentration, pH changes during the extraction process were insignificant.

The transient extraction percentage of bismuth(III) as a function of time for the feed solution adjusted to pH 1.4 was depicted in Fig. S7. The obtained results revealed that 80% of bismuth was extracted in 6 h.

### Effect of sulfate ion concentration

Many common methods for extracting bismuth involve leaching with sulfuric acid. This process generates solutions that contain high concentrations of sulfate ions. Therefore, the effect of sulfate ion concentration on bismuth extraction efficiency using the optimized membrane was investigated. The variation of extraction percentage of bismuth(III) in the aqueous feed phase solutions, containing 0.1, 0.2, 0.5, and 1 mol L^−1^ sodium sulfate, are illustrated in Fig. S8. The obtained results showed that although the presence of sodium sulfate in the aqueous feed phase at concentrations of 0.1 and 0.2 mol L^−1^ had no significant effect on bismuth extraction, at higher concentrations (0.5 and 1 mol L^−1^), it resulted in a decrease in the initial rate and extraction percentage of bismuth. This can be attributed to the formation of bismuth-sulfate complexes and the effect of ionic strength^9,50^. Accordingly, the concentration of sulfate ions in the feed solutions was adjusted to 0.2 mol L^−1^ throughout the subsequent experiments.

### Back-extraction study

The back-extraction study was conducted to determine the proper reagent for the stripping of extracted bismuth from the bismuth(III) loaded optimized membrane. Taking into account that D2EHPA is a cation exchange and acidic extractant, hydrochloric acid and sulfuric acid in the concentration range of 0.5–1 mol L^−1^ were used as the receiving solutions. The back-extraction percentage of bismuth(III) from loaded PIM using hydrochloric acid and sulfuric acid in different concentrations is presented in Fig. S9. As can be observed, the back-extraction efficiency was improved by increasing the acid concentration, regardless of the type of acid used. Additionally, sulfuric acid at a 1 mol L^−1^ concentration was the most efficient stripping reagent, capable of quantitatively back-extracting bismuth ions. This effect was most likely owing to the complexation of bismuth with chloride and sulfate ions, that its complexes with sulfate ions are more stable than chloride complexes^9,51^.

These obtained results align coherently with the proposed explanation regarding the influence of solution acidity on the extraction process (Section “Effect of pH”) where it was observed that when the pH values were less than 1.4, there was a noticeable decline in the extraction efficiency. To determine the appropriate time for the back-extraction process, after selecting sulfuric acid as the suitable stripping reagent, back-extraction experiments were performed as a function of time with concentrations of 0.5, 1, and 1.5 mol L^−1^ of sulfuric acid. The back-extraction percentage of bismuth(III) as a function of time for stripping solutions with different concentrations of sulfuric acid is presented in Fig. S10. The results depicted in Fig. S10 confirmed that increasing the concentration of sulfuric acid from 0.5 to 1 mol L^−1^ raises the back-extraction efficiency, and the quantitative back-extraction of bismuth(III) was achieved for the 1 and 1.5 mol L^−1^ of sulfuric acid in 10 min. It is noteworthy that the independence of back-extraction efficiency on the sulfuric acid concentration in the receiving solution was observed beyond 1 mol L^−1^. So, all the back-extraction experiments were performed from receiving solutions containing 1 mol L^−1^ sulfuric acid.

### Determination of stoichiometry of extracted bismuth(III) complex

It is demonstrated that D2EHPA exists in the dimeric form in PIMs^50^. Hence, the stoichiometry of bismuth(III) extraction by the optimized PIM is given by

1 $$Bi^{3 + }_{aq} + \, n\left( {HR} \right)_{PIM} \rightleftarrows BiR_{m} \left( {HR} \right)_{{\left( {n - m} \right),PIM}} + mH^{ + }_{aq}$$

Also, the extraction equilibrium constant (*K*_*ex*_) can be calculated as

2 $${K}_{ex}=\frac{[\text{Bi}{R}_{m}({HR)}_{(n-m)}{]}_{PIM} {\left[{H}^{+}\right]}_{aq}^{m}}{[{Bi}^{3+}{]}_{aq} {[(HR)]}_{PIM}^{n}}$$

where HR represents the D2EHPA and subscripts (aq) and (PIM) refer to the aqueous phase and PIM, respectively. Assuming that bismuth ions are extracted by D2EHPA as BiR_3_(HR)_3_, and the concentration of the D2EHPA in the PIM was constant in the extraction process, the apparent extraction equilibrium constant (*K*′) can be defined as

3 $${K}^{\prime}={K}_{ex}[\left(HR\right){]}_{0,PIM}^{n}$$

By combining Eqs. (2) and (3), the expression of the distribution coefficient of bismuth(III) as a function of pH) can be derived as follows

4 $$\text{lg}D=\text{lg}\left(\frac{[Bi{L}_{m}({HR)}_{\left(n-m\right)}{]}_{PIM}}{[{Bi}^{3+}{]}_{aq}}\right)= {K}^{\prime}+m\text{pH}$$

**Figure 7:**
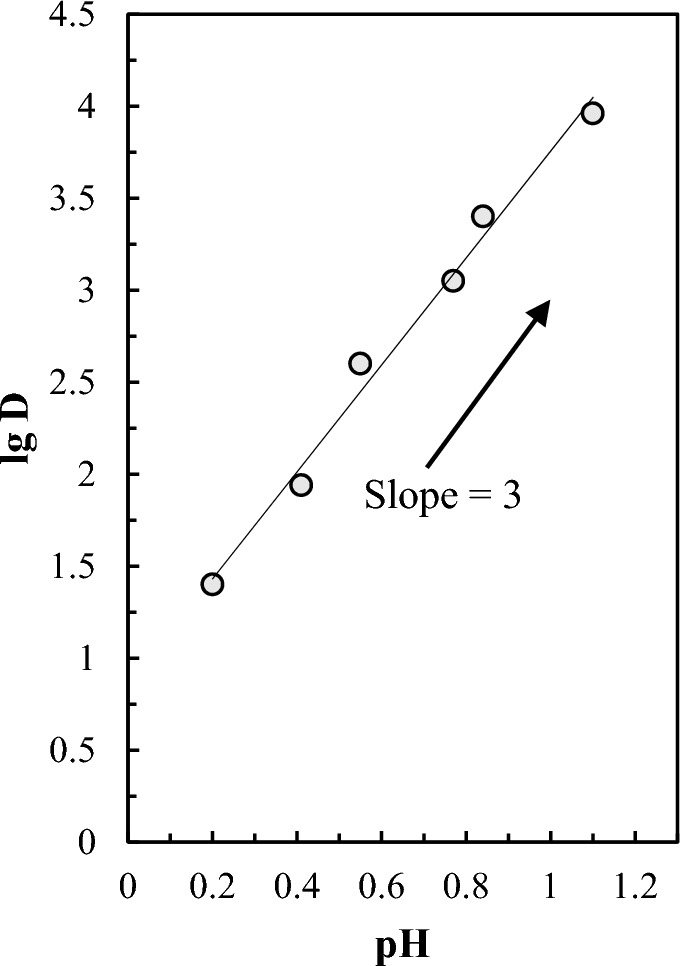
logD versus pH. The experimental conditions are as in Fig. 2.

By linear fitting the experimental data shown in Fig. 6 for pH values ranging from 0.2 to 1.4 to Eq. (4), a slope was obtained as 2.9 (Fig. 7). This slope represents the number of H^+^ ions released in the bismuth extraction process according to Eq. (1), which can be assumed to be equal to 3.

To determine the stoichiometry of the extraction process, which is equal to value n in Eq. (1), extraction experiments were performed by immersing segments of the optimized PIM weighing 0.0500 ± 0.0004 g into 50 mL of feed solutions with bismuth(III) concentrations ranging from 12 to 201 mg L^−1^. After 4 h, the membrane segments were rinsed and immersed in the stripping solutions containing 1 mol L^−1^ sulfuric acid for 10 min, after which the bismuth(III) concentration was measured.

**Figure 8:**
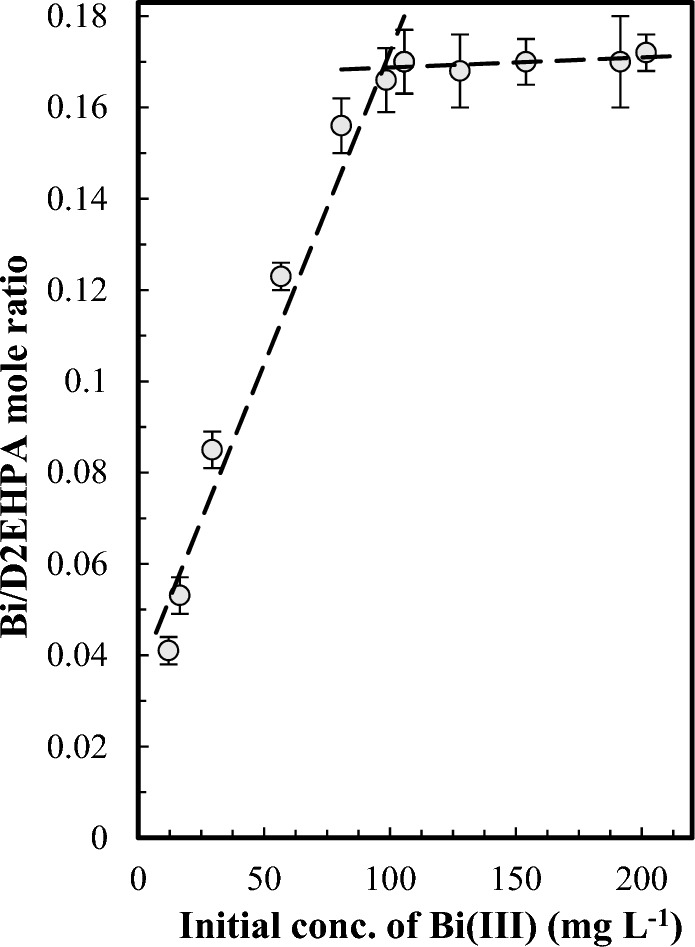
The stoichiometry estimation of bismuth(III) extracted complex in the selected PIM (60/40 wt% PVDF-HFP/D2EHPA) by Job’s method. The experimental conditions are as in Fig. 2.

The changes in the mole ratio of extracted bismuth(III) to the D2EHPA in the optimized membranes as a function of the initial bismuth(III) concentration in the feed solution were presented in Fig. 8. As can be observed, the mole ratio 0.17 was obtained, indicating that the stoichiometry of Bi to D2EHPA in the corresponding complex in the PIM matrix was determined to be 1:6.

The extraction stoichiometry was further investigated using the method introduced by St. John et al.^52^. To this end, extraction experiments were conducted by immersing segments of optimized membranes with different masses into 50 mL feed solutions with a concentration of 20 mg L^−1^ of bismuth(III), adjusted to pH 1.4, for 24 h. The masses of the PIM segments were varied to achieve a mole ratio between D2EHPA in the PIM to bismuth(III) in the feed solution, ranging from 1:1 to 10:1. After reaching equilibrium, the amount of bismuth(III) in the feed solutions was measured. The variation of the mole ratio of bismuth(III) to D2EHPA in the PIM as a function of the mole ratio of the D2EHPA in the PIM to bismuth(III) in the feed solution is presented in Fig. S11. As can be observed, when the mole ratio of the D2EHPA in the membrane to bismuth(III) in the feed solution was lower than 6:1, the PIM segments were fully loaded with bismuth(III). Moreover, when the ratio was greater than 6:1, the number of free D2EHPA in the PIM rapidly increased. The obtained results revealed a 1:6 ratio for the stoichiometry of Bi to D2EHPA in the corresponding complex. These results agree well with the stoichiometric results obtained from the previous method.

The obtained value 3 for n in Eq. (1) and stoichiometry of Bi to D2EHPA suggested that bismuth extracted as Bi^3+^, D2EHPA was as the dimeric form in the PIM, and stoichiometric Bi/D2EPAcomplex was BiL_3_(HL)_3_. The obtained stoichiometry results agree with other studies^39,53,54^. It should be worthy of note that the possible chemical structure of the Bi/D2EHPA complex is shown in Fig. S12.

### Performance of selected PIM in the extraction of bismuth(III) from low-concentration solutions

One of the determining factors of the efficiency of the extraction process is the ability to extract target ions from low-concentration solutions. To perform this investigation, a series of extraction experiments were conducted from feed solutions with various volumes in the range from 5 to 300 mL containing 0.005 mmol bismuth(III) and 0.2 mol L^−1^ sulfate adjusted to pH 1.4. The obtained results are presented in Table S1. The results revealed that increasing the volume of the feed solution decreases the extraction percentage of bismuth(III). Additionally, quantitative back-extraction of bismuth(III) to the stripping solution was observed, which can be utilized for designing a preconcentration method for bismuth recovery.

### Selectivity study

In the solutions used for bismuth recovery, in addition to bismuth ions, various other ions are present. Therefore, the selectivity of the optimized PIM was investigated in the extraction of bismuth(III) from its binary mixtures and a mixture containing all of the ions including Mo(VI), Al(III), Fe(III), Cr(III), Mn(II), Zn(II), Cu(II), Ni(II), Cd(II) and Co(II). The concentration of bismuth(III) and each of the mentioned ions in the binary mixtures and mixture of all ions was 20 mg L^-1^. The obtained results, presented in Table S2, revealed that only Fe(III) co-extracted with bismuth(III), and the presence of other investigated ions did not interfere with the extraction of bismuth(III).

Taking into account that D2EHPA is not a selective extractant, and the selectivity of D2EHPA for the extraction of cations is pH-dependent, among the studied cations, except for Fe(III), the remaining cations are extracted at a pH higher than the optimized pH for the extraction of bismuth(III). So, except for Fe(III), other investigated cations have no interference in the extraction of bismuth(III). Additionally, molybdenum ions exist in anionic forms at optimized pH and can't be extracted by D2EHPA.

Fluoride could create an anionic complex with Fe(III) and act as a masking agent for Fe(III)^55^. Therefore, the effect of adding sodium fluoride to eliminate the interference of Fe(III) was investigated. To this end, the extraction experiments were performed from a feed solution containing a mixture of all the mentioned ions and 0.01 mol L^−1^ sodium fluoride. The extraction and back-extraction percentages of all ions are given in Table S3. The results revealed that the presence of fluoride not only eliminated the interference of Fe(III) also decreased the extraction of other cations.

### Stability study

To evaluate the reusability of the studied PIM, consecutive extraction/back-extraction cycles were performed under optimal conditions. The extraction/back-extraction cycles were continued for up to 15 cycles. The feed and stripping solutions are renewed after each extraction and back-extraction experiment. The results are depicted in Fig. S13. As can be observed, the studied membrane showed great stability with no considerable decrease in the extraction and back-extraction percentages after 15 cycles.

### Application of the studied PVDF-HFP-based PIM

To assess the performance of optimized PIM in the extraction of bismuth(III) from the real sample, under optimized conditions, the optimal PIM was employed for the recovery of bismuth(III) from a zinc electrowinning sludge sample. A zinc production plant in Zanjan province-Iran provided a zinc electrowinning sludge sample. The real sample was leached by 0.25 mol L^−1^ sulfuric acid and then adjusted to pH 1.4. The 0.02 mol L^−1^ NaF was added to eliminate the interference of Fe(III). The obtained results are given in Table S4 These results revealed that the studied PVDF-HFP-based PIM has great potential for extracting bismuth(III) ions from the examined real sample.

## Conclusion

This study illustrated that PVDF-HFP is a proper base polymer for the fabrication of PIMs comprised of D2EHPA as the extractant. The optimal PIM composition for the extraction of bismuth(III) from sulfate media was determined to be 60 wt% PVDF-HFP and 40 wt% D2EHPA. Under optimum conditions, bismuth(III) was extracted effectively from the feed solution containing 0.2 mol L^−1^ sulfate ions and 20 mg L^−1^ bismuth(III) that was adjusted to pH 1.4. Completely back-extraction of bismuth(III) was achieved using 1 mol L^−1^ H_2_SO_4_ as the receiving solution. It was proved that D2EHPA was dimer in the investigated PIM. Two different methods were used to investigate the stoichiometry of the PIM-bismuth(III) complex, and the formation of the D2EHPA-bismuth(III) complex was determined as Bi.R_3_.(HR)_3_, where HR represents D2EHPA. The investigated PVDF-HFP-based PIM showed high selectivity in the extraction of bismuth(III) over other interfering ions such as Mo(VI), Cr(III), Fe(III), Al(III), Ni(II), Zn(II), Cd(II), Co(II), Cu(II), and Mn(II). The interference of Fe(III) was eliminated by masking with fluoride. The newly developed PIM exhibited high stability in 15 sequential extraction/back-extraction cycles. The characterization of optimal PIM by FT-IR revealed that D2EHPA was dispersed in the PVDF-HFP matrix in physical form and without chemical interactions. The cross-section image of PIM illustrated the presence of D2EHPA droplets in the investigated PIM. The stress–strain results confirmed the plasticizer effect of D2EHPA.

### Supplementary Information

Supplementary Information.

## Data Availability

Data underlying the results presented in this paper are not publicly available at this time but may be obtained from Davood Kazemi (corresponding author) upon reasonable request.
