# Supplementary material for: PVDF-HFP-based polymer inclusion membrane functionalized with D2EHPA for the selective extraction of bismuth(III) from sulfate media
Source: Sci Rep. 2024 May 21;14:11622. doi: 10.1038/s41598-024-62401-8 (PMC11109169; doi:10.1038/s41598-024-62401-8)
Supplement: Supplementary file 1 — Supplementary Information. [file 41598_2024_62401_MOESM1_ESM.docx]

**Supplementary Information**

**PVDF-HFP-based polymer inclusion membrane functionalized with D2EHPA for the selective extraction of bismuth(III) from sulfate media**

Davood Kazemi*, Mohammad Reza Yaftian*

*Department of Chemistry, Faculty of Science, The University of Zanjan, 45371-38791 Zanjan, Iran*

*Corresponding authors: Davood Kazemi, Mohammad Reza Yaftian

E-mail address: [d.kazemi.ch@znu.ac.ir](mailto:d.kazemi.ch@znu.ac.ir) (Davood Kazemi), [yaftian@znu.ac.ir](mailto:yaftian@znu.ac.ir) (Mohammad Reza Yaftian)

**Figure S1**


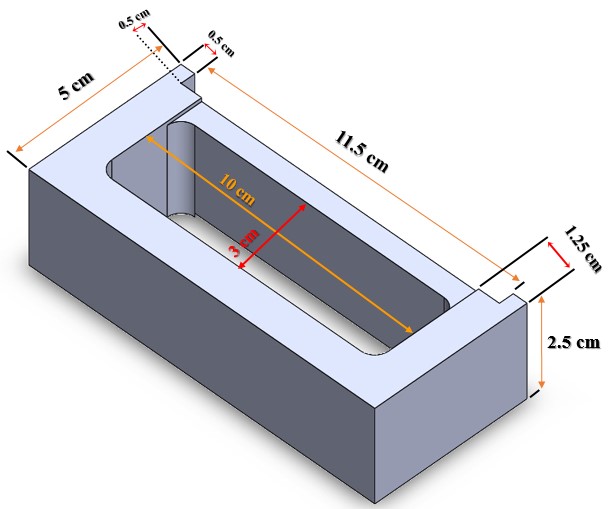

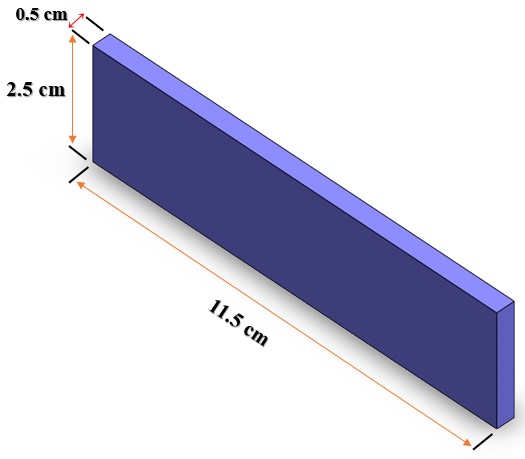


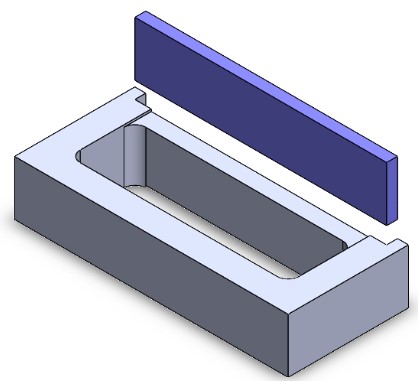

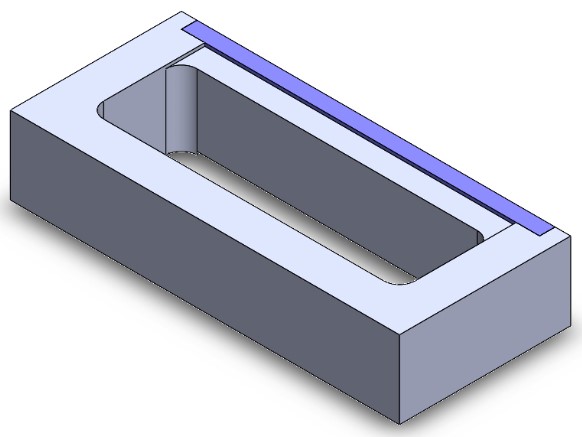


**Figure S1.** Schematic of the casting knife.

**Figure S2**

**Figure S2.** The transient extraction percentage of bismuth(III) by PVDF-HFP-based PIMs with different plasticizers in the PIMs. (PIMs: 60/40 wt% PVDF-HFP/D2EHPA (◊), 55/40/5 wt% PVDF-HFP/D2EHPA/NPOE (○), 55/40/5 wt% PVDF-HFP/D2EHPA/TBP (●), 55/40/5 wt% PVDF-HFP/D2EHPA/DBP (∆), 55/40/5 wt% PVDF-HFP/D2EHPA/TEHP (▲), 55/40/5 wt% PVDF-HFP/D2EHPA/DEHP(), 55/40/5 wt% PVDF-HFP/D2EHPA/1-TD (■)). The experimental conditions are as in Fig. 2.

**Figure S3**

**Figure S3.** The contact angle of blank PVDF-HFP film and selected PIM.

**Figure S4**


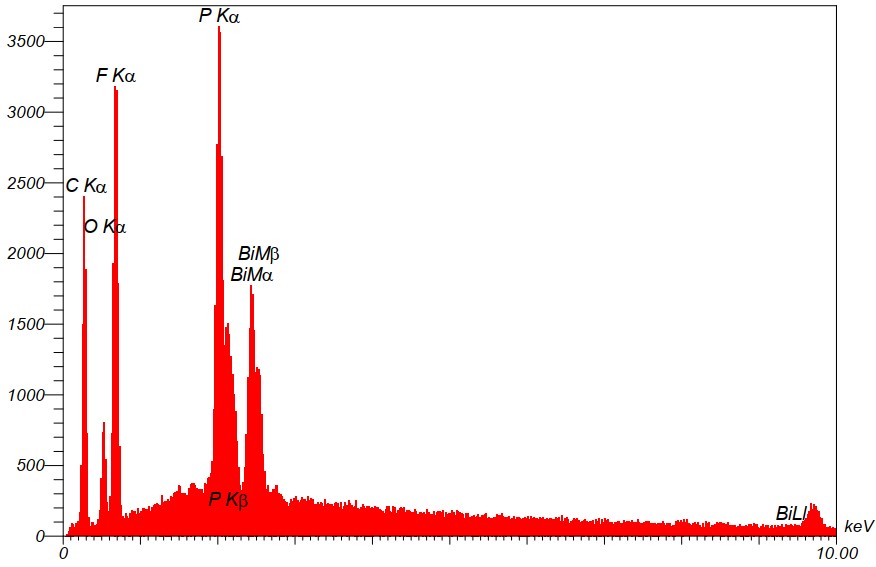


**(a)**


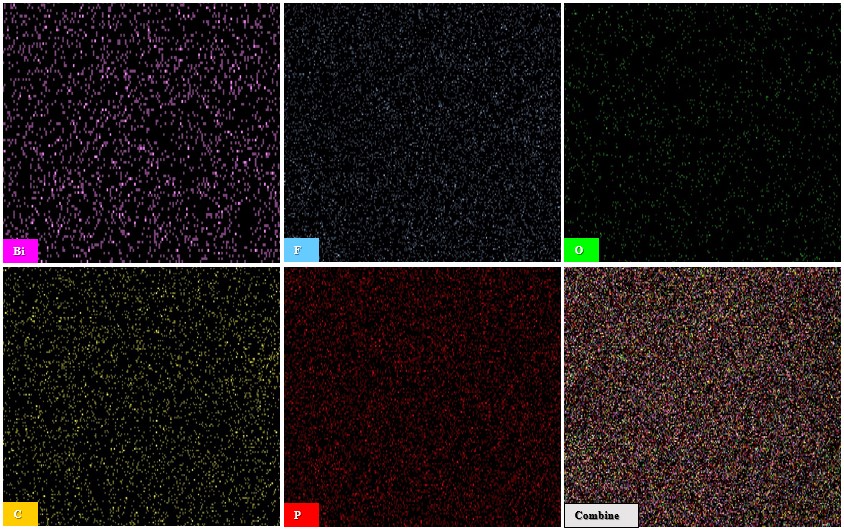


**(b)**

**Figure S4.** EDS spectra of optimized PIM loaded with bismuth(III). (a) EDS spectra of the selected PIM, and (b) EDS mapping images of the selected PIM.

**Figure S5**


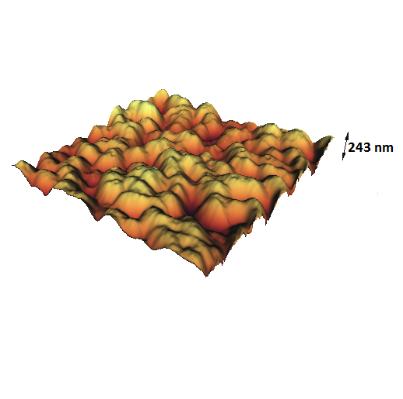

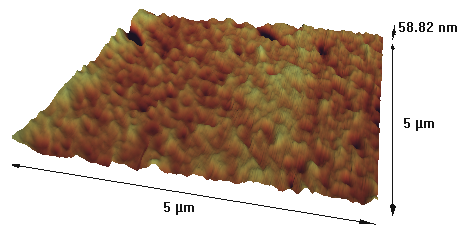


**(a)**

**(b)**

**Figure S5.** 3D-AFM images of (a) blank PVDF-HFP film and (b) selected PIM.

**Figure S6**

**Figure S6.** Stress-strain curves of the (a) blank PVDF-HFP film and selected PIM (60/40 wt% PVDF-HFP/D2EHPA) (b) optimized PIM (60/40 wt% PVDF-HFP/D2EHPA) and PIM composed of 50/50 wt% PVDF-HFP/D2EHPA.

**Figure S7**

**Figure S7.** The transient bismuth(III) extraction curve for the selected PIM in the pH 1.4. The remaining experimental conditions are as in Fig. 2.

**Figure S8**

**Figure S8.** Extraction of bismuth(III) from aqueous feed solutions with different concentrations of sulfate (0.1(⯁), 0.2(□), 0.5 (∆), 1(●) mol L^-1^ sulfate) into the selected PIM. The remaining experimental conditions are as in Fig. 2.

**Figure S9**

**Figure S9.** Back-extraction of bismuth(III) from selected PIMs into the 50 mL of H_2_SO_4_ or HCl stripping solutions with different concentrations. Shaking rate: 200 rpm. Back-extraction time: 6 h. Temperature 25 ± 1^◦^C.Error bars ± standard deviation (SD) (n=3).

**Figure S10**

**Figure S10.** Back-extraction of bismuth(III) from selected PIMs into the 50 mL of H_2_SO_4_ with different concentrations (0.5 (○), 1 (●), 1.5 (▲) mol L^-1^). Back-extraction time: 30 min. The remaining experimental back-extraction conditions are as in Fig. S9.

**Figure S11**

**Figure S11.** The stoichiometry estimation of bismuth(III) extracted complex in the selected PIM (60/40 wt% PVDF-HFP/ D2EHPA) by St. John and his co-workers method. The experimental conditions are as in Fig. 2.

**Figure S12**


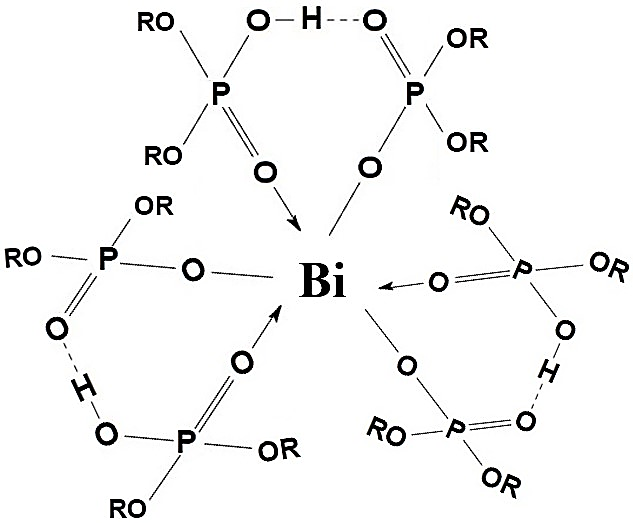


**Figure S12.** Possible chemical structure of the Bi/D2EHPA complex.

**Figure S13**

**Figure S13.** Fifteen consecutive extraction/back-extraction experiments. The transient extraction and back-extraction percentage of bismuth(III) are presented with the black and gray bars, respectively.

**Table S1.** Extraction of bismuth(III) from feed solutions with different volumes.

| Volume (ml) | Initial concentration  (mg L^-1^) | Extraction (%) | Concentration of Bi(III) in the receiving solution |
| --- | --- | --- | --- |
| 50 | 20 | 80 ± 1.1 | 16 ± 0.9 |
| 100 | 10 | 78.2 ± 1.6 | 15.6 ± 1.4 |
| 150 | 7.5 | 65.4 ± 1.3 | 13.1 ± 1.1 |
| 200 | 5 | 52.2 ± 0.4 | 10.4 ± 1.7 |
| 250 | 3.75 | 43.9 ± 1.2 | 8.8 ± 1.2 |
| 300 | 2.5 | 42.5 ± 1.8 | 8.5 ± 1.5 |

**Table S2.** Extraction of bismuth(III) from its binary and multiple mixtures containing common interfering metal ions with selected PIM (60/40 wt% PVDF-HFP/D2EHPA)^a^.

| No. | Mixture | Extraction | Back-extraction |  | No. | Mixture | Extraction | Back-extraction |
| --- | --- | --- | --- | --- | --- | --- | --- | --- |
| 1 | Bi(III)  - | 80.0 ±0.6  - | 80.6 ±1.5  - |  | 8 | Bi(III)  Cd(II) | 79.8 ±0.7  9.3 ±0.8 | 78.9 ±0.4  ND |
|  |  |  |  |  |  |  |  |  |
| 2 | Bi(III)  Mo(VI) | 80.2 ±0.3  1.4 ±0.6 | 80.0 ±0.6  ND^b^ |  | 9 | Bi(III)  Co(III) | 80.2 ±1.7  7.9 ±2.1 | 79.9 ±1.4  ND |
|  |  |  |  |  |  |  |  |  |
| 3 | Bi(III)  Cr(III) | 80.9 ±1.3  4.9 ±0.6 | 79.3 ±0.3  ND |  | 10 | Bi(III)  Cu(III) | 80.4 ±1.4  7.9 ±2.1 | 79.8 ±1.7  ND |
|  |  |  |  |  |  |  |  |  |
| 4 | Bi(III)  Al(III) | 80.8 ±1.9  1.8 ±1.1 | 79.6 ±1.9  ND |  | 11 | Bi(III)  Mn(II) | 80.0 ±1.8  5.9 ±1.1 | 79.8 ±1.2  ND |
|  |  |  |  |  |  |  |  |  |
| 5 | Bi(III)  Fe(III) | 59.0 ±1.5  32.0 ±2.3 | 59.2 ±3.0  15 ±1.4 |  | 12 | Bi(III) | 57.0 ±1.9 | 56.1 ±1.4 |
|  |  |  |  |  |  | Mo(VI) | 1.7 ±0.3 | ND |
|  |  |  |  |  |  | Cr(III) | 4.1 ±0.9 | ND |
|  |  |  |  |  |  | Al(III) | 1.7 ±0.6 | ND |
| 6 | Bi(III)  Ni(II) | 80.8 ±1.9  1.8 ±1.1 | 79.6 ±1.9  ND |  |  | Fe(III) | 30.8 ±1.3 | 14.2 ±2.2 |
|  |  |  |  |  |  | Ni(II) | 6.5 ±1.6 | ND |
|  |  |  |  |  |  | Zn(II) | 4.7 ±0.7 | ND |
|  |  |  |  |  |  | Cd(II) | 7.8 ±2.4 | ND |
| 7 | Bi(III)  Zn(II) | 79.9 ±0.6  5.2 ±1.4 | 80.1 ±0.9  ND |  |  | Co(II) | 4.7 ±2.8 | ND |
|  |  |  |  |  |  | Cu(II) | 2.9 ±1.0 | ND |
|  |  |  |  |  |  | Mn(II) | 5.4 ±1.6 | ND |

^a^Experimental conditions. Extraction experiments; volume and composition of feed solution: 50 mL, 20 mg L^−1^ of each interfering metal ions in 0.2 mol L^-1^ sulfate adjusted to pH 1.4. Back-extraction experiments; volume and composition of stripping solution: 50 mL, 1 mol L^−1^ sulfuric acid. ^b^ND: not detectable.

**Table S3.** Extraction of bismuth(III) from mixtures containing common interfering metal ions and sodium fluoride with selected PIM (60/40 wt% PVDF-HFP/D2EHPA)^a^.

| Mixture | Extraction | Back-extraction |
| --- | --- | --- |
| Bi(III) | 81.0 ± 1.9 | 79.8 ± 2.4 |
| Mo(VI) | ND^b^ | ND |
| Cr(III) | 3.4 ± 1.2 | ND |
| Al(III) | 1.4 ± 0.7 | ND |
| Fe(III) | 3.2 ± 1.8 | ND |
| Ni(II) | 4.1 ± 1.5 | ND |
| Zn(II) | ND | ND |
| Cd(II) | 6.1 ± 2.3 | ND |
| Co(II) | 1.9 ± 0.6 | ND |
| Cu(II) | 1.9 ± 0.7 | ND |
| Mn(II) | 3.7 ± 1.4 | ND |

^a^Experimental conditions such as reported in Table S2, additionally 0.01 mol l^-1^ sodium fluoride in the feed solution. ^b^ND: not detectable.

**Table S4.** Application of the optimized PIM (60/40 wt% PVDF-HFP/D2EHPA) for extraction of bismuth(III) from zinc electrowinning sludge.

| Zinc electrowinning sludge | |  | Leach solution by H_2_SO_4_ |  | Extraction (%) |
| --- | --- | --- | --- | --- | --- |
| Ions | wt% |  | Concentration (mg L^-1^) |  |  |
| Bi | 0.04 ± 0.01 |  | 22.3 ± 1.9 |  | 78.3 ± 1.7 |
| Ni | 0.0014 ± 0.0003 |  | 3.7 ± 0.4 |  | ND^a^ |
| Cd | 0.0007 ± 0.0001 |  | 2.8 ± 0.2 |  | ND |
| Co | 0.0083 ± 0.0003 |  | 14.7 ± 1.6 |  | 1.10 ± 0.04 |
| Fe | 0.1310 ± 0.0007 |  | 40.8 ± 2.9 |  | 3.9 ± 0.7 |
| Mn | 17.90 ± 2.89 |  | 487 ± 32 |  | 4.8 ± 0.6 |
| Zn | 1.41 ± 0.29 |  | 795 ± 19 |  | 4.8 ± 0.8 |
| Pb | 3.82 ± 0.31 |  | 3.1 ± 0.6 |  | ND |
| Cu | 0.0017 ± 0.0004 |  | 1.5 ± 0.2 |  | ND |

^a^ND: not detectable.
